# The Efficacy of Combined Cisplatin and Nanoparticle Albumin-Bound Paclitaxel in a Stage IV Pancreatic Squamous Cell Carcinoma Patient With a Somatic *BRCA2* Mutation: A Case Report

**DOI:** 10.3389/fonc.2021.585983

**Published:** 2021-04-29

**Authors:** Xiaozhun Huang, Chunling Wang, Teng Ma, Zhangkan Huang, Houhong Zhou, Lin Xu, Renjie Zhang, Jianjun Zhao, Yefan Zhang, Zhen Huang, Lin Shao, Yang Wang, Fan Yang, Xu Che

**Affiliations:** ^1^ Department of Hepatobiliary Surgery, National Cancer Center/National Clinical Research Center for Cancer/Cancer Hospital and Shenzhen Hospital, Chinese Academy of Medical Sciences and Peking Union Medical College, Shenzhen, China; ^2^ Department of Hospital Acquired Infection Control, National Cancer Center/National Clinical Research Center for Cancer/Cancer Hospital and Shenzhen Hospital, Chinese Academy of Medical Sciences and Peking Union Medical College, Shenzhen, China; ^3^ Department of Medicine, Burning Rock Biotech, Guangzhou, China; ^4^ Department of Gastrointestinal and Pancreatic Surgery, National Cancer Center/National Clinical Research Center for Cancer/Cancer Hospital, Chinese Academy of Medical Sciences and Peking Union Medical College, Beijing, China

**Keywords:** pancreatic squamous cell carcinoma, cisplatin, nanoparticle albumin-bound paclitaxel, *BRCA2* mutation, overall survival

## Abstract

Pancreatic squamous cell carcinoma (SCC) is a rare primary pancreatic malignancy with a poor prognosis. The median overall survival (OS) for metastatic setting is only 4 months and the optimal management remains poorly defined. In the present study, we report a 52-year-old female patient with stage IV primary SCC of the pancreas harboring a deleteous *BRCA2* somatic mutation. After 10 cycles of chemotherapy of cisplatin combined with nanoparticle albumin-bound paclitaxel, metastatic lesions in the liver and lymph nodes achieved radiographic complete responses and pancreatic lesion shrank from 5.7 to 1.5 cm in diameter. The patient subsequently underwent a posterior radical antegrade modular pancreatosplenectomy with R0 resection and residual liver lesions were also resected. After 3 months, a tumor relapsed in the liver. She was then treated with olaparib combined with pembrolizumab and achieved stable disease on the liver lesion. The patient eventually died from cerebral hemorrhage with a long OS of 21 months. Our case demonstrated a favorable clinical activity and survival advantage of the combined cisplatin and nanoparticle albumin-bound paclitaxel, which might serve as a therapeutic option for the patient with *BRCA*-mutant pancreatic SCC.

## Introduction

Pancreatic squamous cell carcinoma (SCC) is a rare primary pancreatic malignancy, comprising 0.5–5% of all exocrine pancreatic neoplasms. It is characterized by worse survival, largely attributable to that the majority of patients with this entity present with stage IV disease at initial diagnosis (1). The median overall survival (OS) has been reported to range from 7 to 14 months (2–4). In addition, the optimal management for pancreatic SCC remains poorly defined. The initial choice of chemotherapy is extrapolated from experience of pancreatic adenocarcinoma, to which patients often respond poorly (5).

In recent years, greater insight into the molecular biology of pancreatic cancer has prompted new treatment strategies. *BRCA* genes code for proteins that are involved in homologous recombination repair of DNA double-strand breaks (6). Approximately 10% of pancreatic cancers are associated with germline or somatic mutation in *BRCA1* or *BRCA2* (7). Clinical evidence has shown the efficacy and survival advantage of drugs targeting the DNA repair pathway such as platinum-based chemotherapy and PARP inhibitors in germline *BRCA1/2*-mutant patients with pancreatic adenocarcinoma (1, 8). However, little is known about the efficacy of these drugs in *BRCA*-mutant pancreatic SCC owing to its rarity.

Herein, we present a 52-year-old female patient with a stage IV pancreatic SCC harboring a somatic *BRCA2* deleterious mutation, who responded favorably to the chemotherapy of cisplatin and nanoparticle albumin-bound paclitaxel, and achieved a long OS of 21 months.

## Case Description

**Figure 1:**
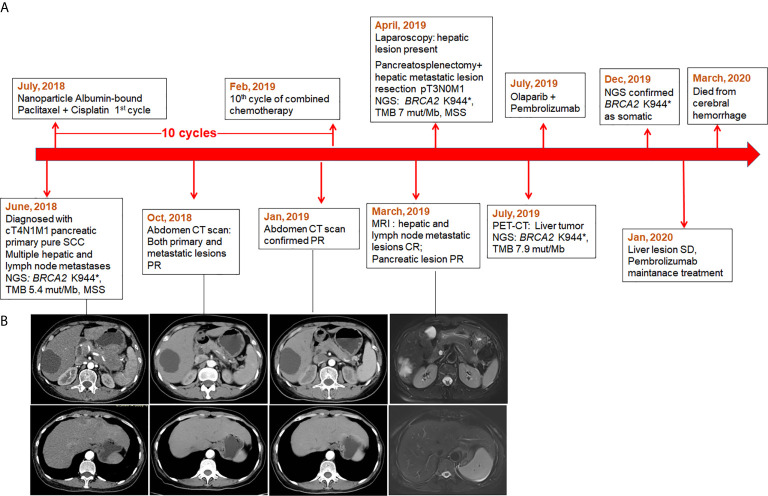
The timeline of patient’s treatment history and the response of the tumor lesions. **(A)** Time line; **(B)** The radiographic imaging of pancreatic and metastatic liver lesions.

**Figure 2:**
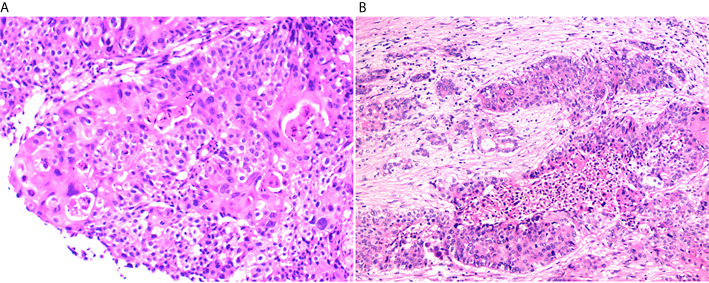
Hematoxylin and eosin (HE) stain of the pancreaticlesion. **(A)** The biopsy of the pancreatic lesion for initial diagnosis indicated squamous cell carcinoma (200×). **(B)** The HE stain of the surgical sample after the combined chemotherapy (100×).

The patient’s clinical course was illustrated in **Figure 1A**. A 52-year-old female patient presented at the hospital in June, 2018 in a good physical condition, complaining of pain in the middle and upper abdomen accompanied by acid reflux and heartburn over the past 5 months. Her serum CA19.9, SCC, and CEA levels were 65.82 U/ml, 25.5 ng/ml, and 11.65 ng/ml, respectively. An abdomen computed tomography (CT) indicated a tumor measuring 7 × 6 cm located in the tail of the pancreas that invaded the posterior wall of the stomach and the splenic artery, accompanied by multiple hepatic and lymph node metastases in the left supraclavicular fossa (**Figure 1B**). Subsequently an endoscopic ultrasound (EUS)-guided biopsy was performed on the pancreatic lesion and the histological and immunohistochemically tests confirmed a cT4N1M1 primary pure squamous cell carcinoma (**Figure 2A**).

A sequencing performed on the biopsied specimen revealed a stop-gain mutation in *BRCA2* exon 11 (c.1830A>T, p.K944*) (**Figure S1**), which has been reported as a pathogenic germline mutation. In addition, the patient had a tumor mutational burden (TMB) of 5.4 mut/Mb and a stable microsatellite status (MSS). Based on the genetic results, a regimen of nanoparticle albumin-bound paclitaxel (125 mg/m^2^) combined with cisplatin (75 mg/m^2^) was eventually decided after a multi-disciplinary panel discussion.

The combined chemotherapy was initiated in July, 2018 (**Figure 1A**). After five cycles of treatment, the patient achieved partial response (PR) on primary pancreatic and metastatic lesions revealed by a total abdomen CT scan (**Figure 1B**) and the serum biomarkers (CA19.9, SCC, and CEA) declined into a normal range. She remained as PR after eight cycles. A magnetic resonance imaging (MRI) was performed in March, 2019 after 10 cycles of combined chemotherapy and showed that both hepatic and lymph node metastases (regional and distanced lymph nodes) achieved complete response (CR) and the primary pancreatic lesion shrank from the initial 5.7 to 1.5 cm in diameter (**Figure 1B**).

Since the imaging examination indicated tumor downstaging, which might be qualified for surgical resection, a preoperative laparoscopy was conducted in April, 2019, and detected multiple nodules on the liver surface. No hepatic space-occupying lesion was detected by the intraoperative US. The nodules on hepatic segments 2, 3, and 4 were resected and sent for histopathological test, and results showed a few malignant tumor cells in the S2 nodule. A laparotomy continued under the request of the patient’s family and revealed a lesion of 4 × 3cm in the pancreatic tail and multiple hepatic lesions. Subsequently, a posterior radical antegrade modular pancreatosplenectomy was performed and hepatic metastatic lesions were also resected. Histological examination of the resected specimens revealed severe tumor degeneration after the combined chemotherapy (**Figure 2B**) with vital tumor cells present only in small areas of primary and liver metastatic lesions. Two of the four peripancreatic lymph nodes had severe degeneration; no residual tumor cells were found in lymph nodes (n = 12). The pathological classification of pT3N0M1 was defined. The sequencing on the tumor tissue showed the retaining of *BRCA2* K944*, a TMB of 8 mut/Mb and MSS.

The patient experienced postoperative thrombocytopenia (platelet account: 6–9 × 10^9^/L) of unknown cause and the platelet account returned to normal (50–60 × 10^9^/L) 3 months later without any treatment. A repeat PET-CT in July, 2019 showed a liver recurrent lesion of 3.3 cm in diameter. The patient was then treated with olaparib (200 mg per day) combined with pembrolizumab (100 mg per 21 days) and achieved stable disease (SD) on her liver lesion 6 months later. The plasma and matched white blood cell samples of the patient were sent for sequencing using a panel with 520-cancer-related genes. The results confirmed *BRCA2* K944* as a somatic mutation, and showed a TMB of 7.98 mut/Mb and MSS status. The patient experienced hepatic artery embolization and severe low platelet count. She died from a cerebral hemorrhage in March, 2020 with an OS of 21 months.

## Discussion

Surgery remains the cornerstone in the management of pancreatic SCC. However, the standard care for the metastatic setting is still lacking (9, 10). Data from sporadic reports revealed that 38.5% of metastatic patients received palliative care, and others commonly underwent a combination of cisplatin and 5‐fluorouracil or gemcitabine, showing a short median OS of 4 months, compared with 17 months in the resectable group and 8 months in the locally advanced tumor group (11). A pool of data regarding pancreatic SCC available in the public domain showed a median OS of 7 months in 54 patients with pancreatic SCC, and resectable cases had significantly better OS when compared with non-resectable cases (10 months *versus* 4 months, respectively) (10). In stage IV disease, a significant difference in median OS was noticed between the patients who received chemoradiation (4.9 months) as compared to that of the patients who did not receive chemoradiation (1.5 months) (12). S. Kumar Das Majumdar et al. reported a case of a locally advanced SCC of the pancreatic tail showing an OS of 1 year following treatment with an albumin-free nanosomal paclitaxel lipid suspension-based regimen (13). A recent study described a patient with T2N0M0R0 primary SCC of the pancreas, who received chemotherapy of gemcitabine following surgery and achieved a long relapse-free survival of 26 months (11).

In the present study, the combined chemotherapy of cisplatin and nanoparticle albumin-bound paclitaxel showed a substantial down-sizing effect in a *BRCA2*-mutant patient with stage IV pancreatic SCC, increased the tumor resectability and significantly prolonged the OS to 21 months, which is the longest survival reported for metastatic pancreatic SCC so far.

NCCN guidance recommends cisplatin combined with gemcitabine for patients with locally advanced or metastatic pancreatic adenocarcinoma for known *BRCA1/2* mutation. However, the *BRCA* mutation in pancreatic SCC has rarely been reported. Schultheis et al. described a locally advanced pancreatic SCC patient with a germline *BRCA2* mutation, who responded favorably to neoadjuvant radiochemotherapy with gemcitabine and obtained an OS of 10 months (14). To the best of our knowledge, our study presents the first BRAC-deficient pancreatic SCC case with a beneficial response to cisplatin-based chemotherapy. In addition, the nanoparticle albumin-bound paclitaxel has been shown to reduce the dense tumor stroma, allowing the chemotherapeutics being delivered to tumor tissue more efficiently (15), which might further explain the tremendously well tumor response in our case.

Despite the preoperative laparoscopy revealed liver metastatic lesions after the combined chemotherapy, the patient underwent radical resection requested by her family and R0 resection was achieved. Postoperatively, she received a regimen of olaparib combined with pembrolizumab after the liver lesion relapsed, and achieved an SD, which suggests a potential efficacy of PARP inhibitors in somatic *BRAC1/2* mutant pancreatic SCC. Although olaparib is only approved for pancreatic adenocarcinoma with germline *BRCA1/2* mutation, the latest study showed comparable response rates and survivals with PARP inhibitors for patients harboring somatic versus germline *BRCA* mutations (16). Our study also provides preliminary evidence for extending the indication to the somatic *BRAC1/2* mutant setting.

In conclusion, we described an exceptional response and survival advantage in a *BRCA2*-mutant patient with metastatic pancreatic SCC who was treated with the combination of cisplatin and nanoparticle albumin-bound paclitaxel. Our data suggest an effective and tolerable therapeutic option for this rare disease entity.

## Data Availability Statement

The original contributions presented in the study are included in the article/**Supplementary Material**. Further inquiries can be directed to the corresponding authors.

## Ethics Statement

Written informed consent was obtained from the individual(s) for publication of any potentially identifiable images or data included in this article.

## Author Contributions

XH, CW, LX, and XC contributed to conception and design of the study. TM, ZkH, HZ, RZ, JZ, YZ, ZH, YW, and FY contributed to the acquisition, analysis, or interpretation of data for the work. LX and LS wrote the first draft of the manuscript. All authors contributed to the article and approved the submitted version.

## Funding

The study was supported by the Sanming Project of Medicine in Shenzhen (No. SZSM202011010).

## Conflict of Interest

The authors declare that the research was conducted in the absence of any commercial or financial relationships that could be construed as a potential conflict of interest.

## Supplementary Material

Supplementary Figure 1 The IGV picture of *BRCA2* exon 11 c.1830A>T, p.K944*
